# MicroRNA-26a-5p as a potential predictive factor for determining the effectiveness of trastuzumab therapy in HER-2 positive breast cancer patients

**DOI:** 10.37796/2211-8039.1150

**Published:** 2021-06-01

**Authors:** Sadra Samavarchi Tehrani, Ehsan Zaboli, Farzin Sadeghi, Soraya Khafri, Ansar Karimian, Mahnoosh Rafie, Hadi Parsian

**Affiliations:** a Student Research Committee, Babol University of Medical Sciences, Babol, Iran; b Cellular and Molecular Biology Research Center, Health Research Institute, Babol University of Medical Sciences, Babol, Iran; c Gastrointestinal Cancer Research Center, Mazandaran University of Medical Sciences, Sari, Iran; d Department of Epidemiology, Babol University of Medical Sciences, Babol, Iran

**Keywords:** Trastuzumab, Real-time PCR, MicroRNA-26a, HER-2 positive, Breast cancer

## Abstract

**Background:**

Breast cancer (BC) is known as the most prevalent type of cancer among women. Trastuzumab, as an anticancer drug, has been used broadly in human epidermal growth factor receptor 2 (HER-2) positive (+) BC patients. Moreover, accumulating evidence has demonstrated that microRNAs is involved in the pathogenesis BC. Hence, we aimed to investigate the effect of trastuzumab on the expression levels of microRNA-26a in HER-2 positive BC patients.

**Methods:**

This study was conducted among HER-2 + and HER-2 Negative (−) BC patients. Serum expression of microRNA-26a was detected by real-time PCR. Then, we assessed the correlations of microRNA-26a levels with multiple clinico-pathological characteristics of BC.

**Results:**

In HER-2 + patients, the microRNA-26a expression significantly increased after treatment with Docetaxel/Trastuzumab in comparison to before the treatment levels (p.value = 0.01). However, this overexpression in HER-2-patients after treatment with Docetaxel was not significant compared to the levels before the treatment (p.value = 0.14). In addition, the expression of microRNA —26a has significantly increased in HER-2 + patients who were ≤48 years old and premenopausal after the treatment with Docetaxel/Trastuzumab when compared to the levels before the treatment (p.value = 0.039 vs. 0.031, respectively). Furthermore, there was a significant correlation between the expression of microRNA —26a and the tumor size, stage, estrogen receptor (ER) and progesterone receptor (PR) status in the HER-2 + group before and after the treatment (p.value = 0.043, 0.042, 0.049 and 0.034 respectively).

**Conclusions:**

Trastuzumab led to overexpression of microRNA-26a in HER-2 + BC patients. It seems that the detecting microRNA —26a expression levels, during or after the trastuzumab therapy could be a useful biomarker for monitoring the therapeutic response in HER-2 + BC patients. However, further studies on large populations of women with HER-2+ BC are needed to explore this possible novel biomarker, in more detail, within various clinical contexts.

## Introduction

Breast cancer (BC) is regarded as the most prevalent form of cancer among women in both developed and developing countries, with a high mortality rate worldwide [1]. It was found that approximately 20% of invasive BC cases amplify human epidermal growth factor receptor 2 (HER-2/NEU/c-ERBB2) [2]. HER-2 receptor is a member of the epidermal growth factor receptor (EGFR) family, and its overexpression is associated with proliferation, metastasis, and poor patient’s prognosis [3]. In clinical settings, HER-2+ BC patients are treated with trastuzumab, which is an anti-HER-2 humanized monoclonal antibody [4,5]. Emerging evidence has demonstrated that trastuzumab mediates its therapeutic effects mainly through two mechanisms: I) induction of antibody-dependent cell-mediated cytotoxicity (ADCC), and II) blockage of HER-2 homo- or hetero-dimerization and downstream signaling pathways such as phosphatidylinositol 3-kinase (PI3K)/AKT/mTOR and RAS/RAF/MEK/MAP kinase (MAPK) [6,7]. Despite the reasonable efficacy rate of this drug, the response rate to trastuzumab monotherapy is approximately 30%, and about 60% for regimens combining trastuzumab with other chemotherapy drugs [8,9]. In addition, most patients develop acquired resistance to this drug within one year [10], and unfortunately there are no specific biomarkers for determining the response rate and potential development of acquired resistance to this drug.

MicroRNAs (miRNAs) which are considered to be key members of non-codingRNAs family, are short and single-stranded RNA molecules with a size of 18–24 nucleotide, which play different roles in various cellular events by binding 3′-UTR of target mRNA for the suppression of translation [11–13]. It was found that miRNAs can be measured in different biological body fluids including serum, plasma, and urine [14], and are not only resistant to nucleolytic degradations, but also stable against harsh conditions [15]. Hence, miRNAs can be used as a novel diagnostic and prognostic biomarkers for various diseases, and are able to serve as predictive biomarkers for treatment of neurodegenerative diseases [16], leukemia [17], and various types of cancers, particularly BC [18]. Moreover, changes in the expression miRNAs levels in cancer cells have been related with prognosis and response to target therapies such as trastuzumab [19,20]. In addition, an increased expression level of miRNA-194 as well as blocked cell migration has been demonstrated in BC cell lines of those treated with trastuzumab, which shows the effect of tratuzumab on miRNA-194 expression, and its anti-tumor activity on HER2-overexpressing BC cell lines [21]. Interestingly, miRNA-26a, as a tumor suppressor miRNA, is downregulated in BC cell lines. In addition, its upregulation induces apoptosis by metadherin (MTDH), enhancer of zeste homolog 2 (EZH2) and myeloid cell leukemia 1 (MCL-1) [22,23]. Besides, in BC cell lines, trastuzumab affects the expression miRNA-26a and miRNA-30b levels, and these miRNAs play a vital role in trastuzumab therapy by targeting cyclin E2 [24].

There are only few studies in the literature about the critical role of miRNAs and their dysregulation in target monotherapy with trastuzumab in biological fluids such as serum, plasma, and urine in BC patients. Moreover, to the best of our knowledge, no valid non-invasive biomarkers have ever been found for determining the response to targeted monotherapy with trastuzumab. Therefore, we assessed the effects of trastuzumab on the serum miRNA-26a expression levels in HER-2+ BC patients with the aim of introducing a reliable, innovative biomarker candidate, to be used in the clinical settings.

## 2. Materials and methods

### 2.1. Study design and sample collection

The present study was conducted among 45 patients with BC, who had been recruited from March 2018 to March 2019, to the Imam Khomeini Hospital in Mazandaran, Iran (this is the main oncology center for admissions of BC patients in Mazandaran). BC diagnosis was confirmed, based on clinical examination and histopathology tests performed by an expert pathologist. The study protocol was reviewed and approved by the local Ethical Committee at our institution (1396.65), and a written Informed Consent was obtained from all the patients.

Inclusion criteria were as follows: BC patients with immunohistochemical (ISH) staining for HER-2 protein of 3+ intensity, or amplification of the *HER2* gene on fluorescence in situ hybridization (FISH), and no pervious therapy with trastuzumab. Exclusion criteria were prior history of cardiovascular disease (CVD), chronic kidney disease (CKD), diabetes mellitus (DM), rheumatoid arthritis (RA), and any type of cancer.

All the patients underwent a histological examination and a blood sample was collected from each of them. The participants were classified as HER-2+ BC or HER-2− BC, and were subsequently divided into two subgroups: I) HER-2 positive (+) BC (the study group – treated with Trastuzumab), including 24 patients, and II) HER-2 negative (−) BC (the control group – not treated with Trastuzumab), including 21 patients.

All HER-2+ BC patients received 4 cycles of tri-weekly doxorubicin (Adriamycin, 60 mg/m2) and Cyclophosphamide (600 mg/m2), followed by 4 cycles of tri-weekly Docetaxel (taxotere, 75 mg/m2) plus Trastuzumab (Herceptin, 6 mg/m2). Each patient in the HER-2 – group (control) received 4 cycles of tri-weekly doxorubicin (60 mg/m2) and Cyclophosphamide (600 mg/m2) (similarly to the HER-2+ BC group). However, after receiving these two drugs, they received Docetaxel (75 100 mg/m2) only (without Trastuzumab), according to The National Comprehensive Cancer Network NCCN guidelines (http://www.nccn.org/). 5 mL blood sample was taken from each participant after signing the Informed Consent.

In the HER-2+ BC group, blood samples from 24 patients were obtained before administering Docetaxel (75 mg/m2) plus Trastuzumab (6 mg/m2) (at the beginning of the 5th course). The subsequent (follow-up) blood samples from these 24 patients were obtained after the administration of these two drugs (at the beginning of the 9th course).

**Fig 1:**
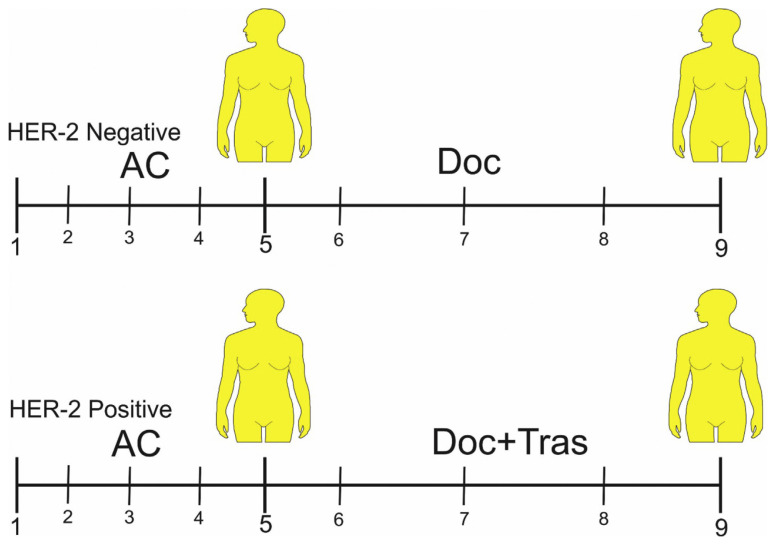
Blood sampling model. Abbreviation: AC (Adriamycin = 60 mg/m^2^ and Cyclophosphamide = 600 mg/m^2^, DOC (Docetaxel = 75 mg/m^2^), Tras (Trastuzumab = 6 mg/m^2^).

In the HER-2− BC group, blood samples from 21 patients were obtained before administering Docetaxel (75 mg/m2) (at the beginning of the 5th course). The subsequent (follow-up) blood samples from these 21 patients were obtained after the administration of this drug (at the beginning of the 9th course) (Fig. 1).

#### Blood sampling

5 mL blood sample was taken from each participant. All of Blood samples were centrifuged at 2500g for 10 minutes at 4°C. Furthermore, serum samples were transferred to 2 mL microtubes and centrifuged for a second time at 12,000 g for 10 minutes at 4°C. The serum samples were transferred at −80°C until final analysis.

### 2.2. Isolation of RNA

Total RNA was extracted from the serum of patients by RiboEX LS (GeneAll, Seoul). 600 μL RiboEX LS Reagent was added to 200 μL serum, and was incubated for 5 minutes at room temperature (25°C), then, 160 μL of chloroform was added and vigorously shaken, and the samples were centrifuged at 12000 g for 15 minutes at 4°C. Furthermore, supernatants were mixed with 400 μL of isopropanol and incubated overnight at −20°C. After centrifuging at 12000 g for 1 hour at 4°C, the resulting pellets were washed with 75% ethanol and centrifuged at 7500g for 5 minutes at 4°C. After allowing the ethanol to completely remove, the pellets were dissolved in 25 μL of diethylenepyrocarbonate-treated water (DEPC-treated water) (SinaClon. Iran). The quality/quantity and purity of RNA was analyzed by optical density measurement with NanoDrop.

### 2.3. Synthesis of specific cDNA

Synthesis of complementary DNA (cDNA) was done by specific stem-loop primers for microR-26a-5p and microRNA-16. In brief, cDNA synthesis was conducted in a 10 μl reaction mixture: 160 ng of total RNA, 5pmol/μl of each stem-loop primers (0.5 μl), 2 μl of 5X buffer (Yekta Tajhiz Azma ®, Iran), 1 μl of each dNTP (Metabion, Germany), 0.5 μl of RNase inhibitor (Metabion, Germany), and 100U (0.5 μl) of MMLVR (Yekta Tajhiz Azma ®, Iran). RNA was reverse transcribed to cDNA with the following conditions: the mixture was incubated for 30 min at 16 °C, at 42 °C for 30 min, and at 72 °C for 10 min. Synthesis of cDNA was done using a peqSTAR 2X Gradient Thermocycler (PEQLAB Biotechnologies GMBH, Erlangen, Germany).

### 2.4. Quantitative real-time polymerase chain reaction (qRT-PCR)

Real-time PCR was done by SYBR Premix Ex TaqTM II (Takara, Japan) to analyze the microRNA-26a expression as a target gene and microRNA016 was used as endogenous control, implemented in the real-time PCR cycler RotorGene Q (QIAGEN GmbH, Hilden, Germany). Briefly, The qRT-PCR was perfomed in a volume of 20 μl containing: A 20 μl real-time PCR reaction mixture consisted of 1 μl cDNA, 10 μl Maxima SYBR-Green/ROX qPCR Master Mix 2X (Ampliqon, Bie & Berntsen, Herlev, Denmark), and 5 pmol/μl of each primer (Table 1). For amplification of the target miRNAs, the PCR cycling conditions were as follows: 15 minutes at 95 °C, followed by 40 cycles of 12 s at 95 °C, and 25 s at 60 °C. The expression level of microRNA-26a-5p was normalized to microRNA-16 by the relative expression software tool.

### 2.5. Statistical analysis

In this study, we used the Shapiro-Wilk test for determining normal distribution of expression microRNA-26a-5p level. In addition, Wilcoxon test was perfomed for comparing the paired serum samples before and after treatment in HER-2^+^ and HER-2^−^ BC patients. Besides, we performed Wilcoxon test to evaluate the effect of clinicopathological characteristics before and after treatment in HER-2 positive patients.

## 3. Results

A total of 45 (24 HER-2^+^ and 21 HER-2^−^) patients participated for determining the expression micro-RNA-26a-5p status before and after Docetaxel therapy in HER-2^−^ group and Docetaxel/Trastuzumab therapy in HER-2^+^ group. All the clinicopathological features of the 45 BC patients are shown in Table 2. In addition, age in HER-2^+^ patients was not significantly different from the HER-2^−^ patients (HER-2^+^ and HER-2^−^: Mean ± SD (49.9 ± 10.1), (51.1 ± 11.2), respectively).

### 3.1. Comparison of serum expression microRNA-26a-5p levels in HER-2^+^ and HER-2^−^ BC patients

**Fig 2:**
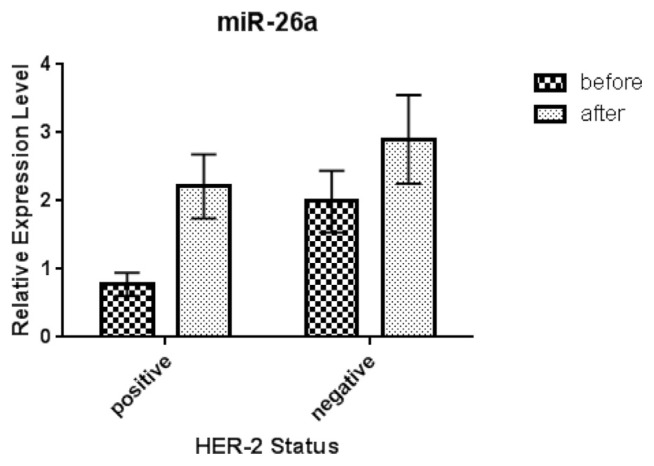
Relative expression of serum miR-26a-5p in HER-2 negative and positive breast cancer patients before and after treatment.

The serum expression microRNA −26a-5p levels in HER-2^−^ patients after treatment with Docetaxel increased in crude data as compared with its expression levels before treatment (not significant, p.value = 0.141). In HER-2^+^ patients, the expression microRNA-26a-5p status significantly increased after treatment with Docetaxel/Trastuzumab in comparison with before treatment (p.value = 0.012) (Fig. 2). In addition, were not any observed significant differences in expression microRNA-26a-5p levels before treatment with Docetaxel in both groups (HER-2 positive and HER-2^−^) (p.value > 0.05). Moreover, we observed a significant difference in expression of microRNA-26a-5p in HER-2^−^ and HER-2^+^ patients after treatment (p.value < 0.05).

To evaluate the effect of menopausal status on the expression microRNA-26a-5p levels, patients were divided into two groups: premenopausal and postmenopausal. The expression microRNA-26a-5p status was significantly increased in HER-2^+^ premenopausal women after treatment with Docetaxel/Trastuzumab when compared to before treatment (p.value = 0.031). However, in postmenopausal women, there was no significant difference in microRNA-26a-5p expression levels before and after treatment with Docetaxel/Trastuzumab in HER-2^+^ patients (p.value = 0.263). In addition, to assess the impact of ageing on the serum expression levels of microRNA-26a-5p, all of the HER-2^+^ patients were categorized into two groups, i.e. ≥ 48 years and >48 years. The results showed that in HER-2^+^ patients, expression microRNA-26a-5p levels were significantly higher in patients ≥48 years after treatment with Docetaxel/Trastuzumab compared to before treatment (p.value = 0.039), whereas in >48 years HER-2^+^ patients, there was no significantly difference before and after treatment with the mentioned drugs (p.value = 0.273) (Table 3).

### 3.2. Relationship between expression microRNA-26a-5p levels and clinicopathological features before and after in HER-2^+^ patients

**Fig 3:**
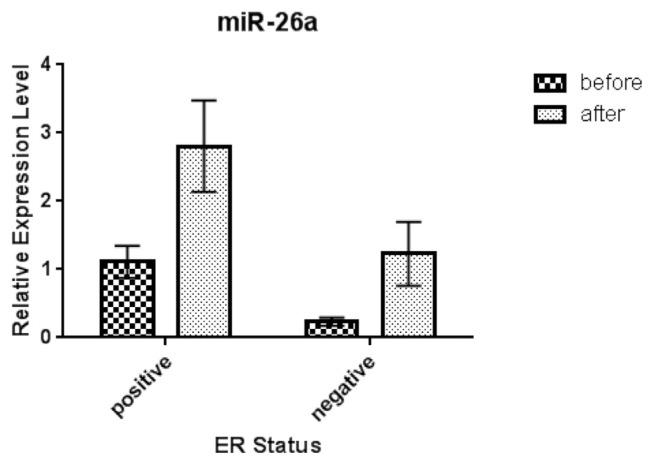
Relative expression of serum miR-26a-5p in HER-2 positive/ER Positive and HER-2 positive/ER Negative breast cancer patients before and after treatment. Abbreviation: ER: esterogene receptor.

**Fig 4:**
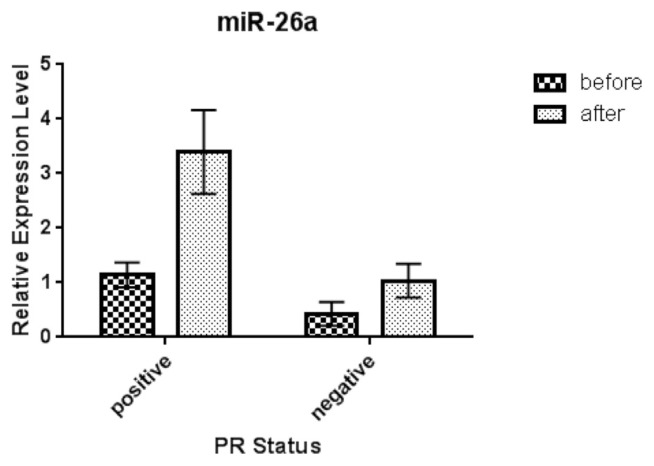
Relative expression of serum miR-26a-5p in HER-2 positive/PR Positive and HER-2 positive/PR Negative breast cancer patients before and after treatment. Abbreviation: PR: progesterone receptor.

In Table 4, clinicopathological features of HER-2^+^ BC patients are shown. As can be seen, there is a significant relationship between expression micro-RNA-26a-5p levels and tumor size (pT3) before and after treatment with Docetaxel/Trastuzumab (p.value = 0.043). Besides, according to histological staging, we observed that there was a significant difference between serum microRNA-26a-5p levels with stage III, before and after treatment in HER-2 positive group (p.value = 0.042). Furthermore, according to the status of ER and PR, expression levels of microRNA-26a-5p had a significant association with ER negative and PR positive before and after treatment in HER-2^+^ individuals (p.value = 0.049 and 0.034, respectively) (Figs. 3 and 4, respectively).

## 4. Discussion

BC is the most prevalent noncoutaneous malignancy among women [25,26]. Based on its etiology, both genetic and environmental factors have a crucial role in the incidence of this malignancy [27,28]. One of the most important genetic factors are MiRNAs, as multifunctional ncRNAs, that have an important function in the consistency and translational efficacy of target RNAs [29–31].

**Fig 5:**
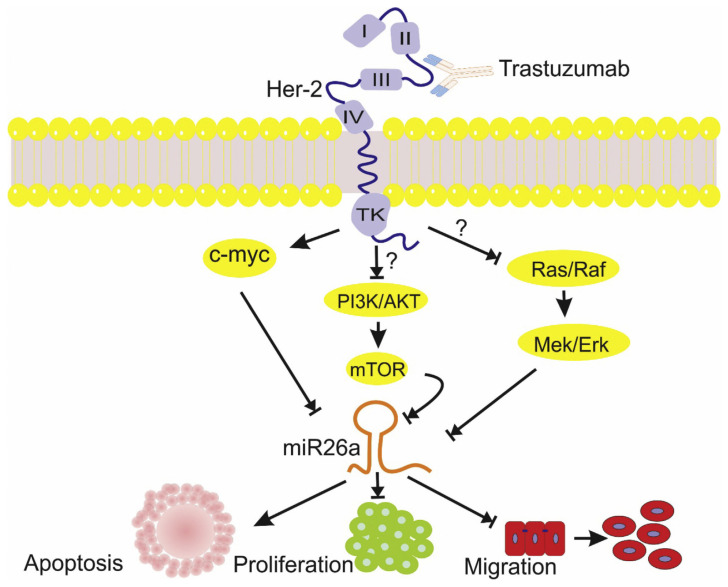
The schematic representation of the effects of trastuzumab on the HER-2 receptor in breast cancer cells. This schematic representation shows the effect of trastuzumab on the HER-2 receptor in breast cancer. Based on our knowledge, the miR-26a inhibits cancer proliferation, migration, and is able to induce apoptosis. Multiple signaling pathways, such C-MYC, PI3K/AKT, and RAS/RAF can repress the expression levels of miR-26a. However, in the HER-2 + BC patients, trastuzumab therapy reduces the levels of these signaling pathways, and thus, increases the miR-26a levels. (The question mark means that the exact mechanism of action is still unknown).

Our data have revealed the upregulation of microRNA-26a in patients with HER-2+ BC, who were treated with trastuzumab. Based on previous studies, the microRNA-26a as a tumor suppressormiR has been reduced in all BC cell lines, and in clinical specimens. Moreover, the expression of microRNA-26a is significantly correlated with the HER-2 status of patients with BC [29,24]. As mentioned, this upregulation occurs in trastuzumab sensitive cell lines but not in acquired or innately resistant trastuzumab cell lines such as BT474r and HCC1954 [32]. Interestingly, upregulation of microRNA-26a was not seen in HER-2− BC patients in our study and these results are consistent with other studies that showed the downregulation of microRNA-26a in triple-negative BC (TNBC) [33]. Recently, growing evidence has revealed that microRNA-26a inhibits cancer proliferation and migration, and is able to induce cell apoptosis. Reduction in the microRNA-26a expression may be due to the numerous regulatory events, such as LOH at chromosome 3p22, mutation in p53 that enhances the post-transcriptional maturation of microRNA-26a, and downregulation of DICER that play a central role in the miRNAs maturation [34–36]. It is not clear how trastuzumab can upregulate the microRNA-26a expression, but a number of mechanisms may explain this regulation (Fig. 5). One possible mechanism is its regulation by MYC. As we know, C-MYC is able to bind transcriptional start sites of the microRNA-26a and repress the expression of this miRNA. MYC is located in the downstream of the HER-2 signaling pathway. Thereby, trastuzumab therapy of HER-2 + BC patients reduces the MYC levels and subsequently increases the microRNA-26a level [37,38]. Besides, trastuzumab inhibits growth factor signaling cascades (including PI3K/AKT/mTOR and RAS/RAF/MEK/MAK kinase) in the downstream pathway of HER-2 by dimerization of HER2. It seems that reducing of the expression levels of these oncogenic factors can lead to an increase in the microRNA-26a, but a confirmation of this hypothesis needs additional studies [39,40].

Taken together, we introduced microRNA-26a, in form of tumor suppressive miRNA in BC based on the clinico-pathological variables, assessed in the present study. In addition, the serum expression of microRNA-26a increased in patients who were in pT3 (TNM staging system) after trastuzumab therapy, and these results are consistent with other studies in which microRNA-26a was upregulated in non-TNBC compared with TNBC cases [33]. Upregulation of miR-26a in BC patients with T3–T4 has been much higher, compared to those with T1–T2 [33]. By contrast, it was reported that miR-26a were overexpressed 3 times and 1.5 times correspondingly in TNBC relative to non-TNBC tumors [41]. Moreover, we observed that expression the micro-RNA-26a levels were correlated with the tumor stage and this microRNA was overexpressed in stage III of HER-2+ BC patients after trastuzumab therapy. In addition, our results have revealed that HER-2+ BC patients with primary tumors had low expression microRNA-26a levels compared to the advanced stages, and this is consistent with previous published study [42]. For instance, it has been shown that miR-26a has different expressions in various stages of gastric cancer: in stage I/II the expression of microRNA-26a was 37% and in stage III/IV this rate increased to 77% [43]. We have not observed any correlation between the expression of microRNA-26a and lymph node metastasis, and it seems that the microRNA-26a probably cannot play a key role in the inhibition of lymph node metastasis. Studies have shown that there are multiple genes as potential downstream target genes of microRNA-26a, which have important functions in tumor growth and metastasis. These include *Prime time entertainment network (PTEN), metadherin (MTDH), cell division protein kinase 6 (CDK6), enhancer of zeste homolog 2 (EZH2), myeloid cell leukemia 1 (MCL-1), Pyruvate Dehydrogenase Complex Component X (PDHX), Chromodomain-helicase-DNA-binding protein 1(CHD1), Growth regulation by estrogen in breast cancer 1 (GREB1), and Karyopherin Alpha 2 (KPNA2)* [33,44]. *MTDH* overexpression, for instance, is associated with the invasion and metastasis in BC, and it has been suggested that the molecular mechanism of this metastasis is related to the induction of the epithelial mesenchymal transition (EMT) in BC and microRNA-26a can inhibit the mRNA and protein levels of *MTDH* [33,45].

Consequently, we provided evidence that micro-RNA-26a increases in ER-/HER-2+ compared with ER+/HER-2 + BC after trastuzumab therapy. According to other studies on ER + BC, with tamoxifen resistance, the reduced level of microRNA-26a has been responsible for this resistance in ER + patients [46]. In addition, our work showed that the micro-RNA-26a is upregulated in PR +/HER-2+ patients, compared with PR-/HER-2+ patients, after treatment with trastuzumab. This upregulation can be independently due to the effect of trastuzumab, but it is the microRNA-26a that regulates the encoding gene of progesterone receptor (PR) [47].

In our study, we have observed an increase in the microRNA-26a expression levels, among premenopausal and ≤48 years old HER-2 + BC patients, after trastuzumab therapy. According to the published studies, HER-2 expression is associated with clinicopathological features, like menopausal status [48,33]. As we know, serum concentration of estrogen is generally evaluated in premenopausal women, compared with postmenopausal ones. In addition, estrogen can induce expression of some growth factors, encoding genes related to various receptors, such as HER-2 [49]. Consequently, expression levels microRNA-26a in premenopausal HER-2+ BC patients are induced after the treatment with trastuzumab.

This study has important strengths that include: 1) providing evidence demonstrating that the miR-26a levels (measured in clinical specimens) are susceptible to modulation by trastuzumab, 2) presenting initial results of the miR-26a analysis, and comparing it with the follow-up findings, in patients with HER-2+ BC and HER-2− BC. However, further large-scale studies are required to confirm our hypothesis. Limitations of our study include a small sample size, short time period of the study followup, difficulties to access clinical data of the patients, and loss of communication with some patients during the follow-up.

Overall, future research with regard to potential predictive biomarkers for trastuzumab targeted therapy effects is needed, in patients with HER-2 + BC. Hopefully, in the future, the miR-26a assessment would help the medical–oncology teams more accurately predict, which patients may have the best chance to positively respond to the treatment with trastuzumab.

## 5. Conclusion

Trastuzumab therapy increases the expression of microRNA-26a in HER-2+ BC patients. Hence, evaluating microRNA-26a levels during the trastuzumab therapy could be considered as a useful biomarker for monitoring the response to treatment in patients with HER-2+ BC. However, further studies on large populations of women with HER-2+ BC are needed to explore this possible novel biomarker, in more detail, within various clinical contexts.

## Figures and Tables

**Table 1:** The primer sequences for cDNA synthesis and quantitative real-time PCR (qRT-PCR).

miRBase accession no.	Primer name	Sequence(5′3′)
MIMAT0000082	has-miR-26a-5p-RT^1^	GTCGTATCCAGTGCAGGGTCCGAGGTATTCGCACTGGATACGACAGCCT
	has-miR-26a-5p-Forward	CGCCGCTTCAAGTAATCCAGG
MIMAT0000069	has-miR-16-5p-RT^1^	GTCGTATCCAGTGCAGGGTCCGAGGTATTCGCACTGGATACGACCGCCAAT
	has-miR-16-5p-Forward	GGTAGCAGCACGTAAATATTGGCG
	Universal- reverse (UR)	GTGCAGGGTCCGAGGT

1 Stem-loop primer for cDNA synthesis

**Table 2:** Characteristics of included HER-2 negative and positive breast cancer patients.

Variable		HER-2 Negative breast cancer N (%)	HER-2 positive breast cancer N (%)
Age (Mean ± SD)		(51.1 ± 11.2)	(49.9 ± 10.1)
ER status	Positive	15 (71.4)	15 (62.5)
	Negative	6 (28.6)	9 (37.5)
PR status	Positive	15 (71.4)	12 (50.0)
	Negative	6 (28.6)	12 (50.0)
Tumor size	pT1	6 (28.6)	5 (20.8)
	pT2	13 (61.9)	14 (58.3)
	pT3	2 (9.5)	5 (20.8)
Lymph nodes	N0	9 (42.9)	12 (50.0)
	N1	7 (33.3)	8 (33.3)
	N2	3 (14.3)	4 (16.7)
	N3	2 (9.5)	0 (0.0)
Tumor stage	I	2 (9.5)	7 (29.2)
	II	12 (57.1)	12 (50.0)
	III	7 (33.3)	5 (20.8)
Tumor grade	I	1)4.8)	3 (12.5)
	II	16 (76.2)	18 (75.0)
	III	4 (19.2)	3 (12.5)
Histological subtype	IDC	17 (81.0)	24 (100.0)
	ILC	4 (19.0)	0 (0.0)
Menopausal status	Premenopausal	11 (52.4)	16 (66.6)
	Postmenopausal	10 (47.6)	8 (33.3)

**Abbreviation**: ER: esterogene receptor, PR: progesterone receptor.

**Table 3:** Comparison of serum level of miR-26a-5p based on menopausal status and age category in HER-2 positive patient before and after treatment.

Variable		Groups		
		
		Before HER-2 positive Mean ± SE (Median)	After HER-2 positive Mean ± SE (Median)	p. value
Menopausal status	Premenopausal	0.603 ± 0.197 (0.248)	1.971 ± 0.527 (0.986)	0.031
	Postmenopausal	1.145 ± 0.312 (1.212)	2.706 ± 0.987 (1.452)	0.263
Age status	≤48	0.643 ± 0.189 (0.255)	1.921 ± 0.498 (1.118)	0.039
	48<	1.127 ± 0.359 (1.148)	2.933 ± 1.1091 (1.469)	0.237

**Table 4:** Characteristics of breast cancer patients and the relationship between serum levels of miR-26a-5p and clinicopathological parameters of HER-2 positive breast cancer patients.

Variable		Groups		
		
		Before HER-2 positive Mean ± SE (Median)	After HER-2 positive Mean ± SE (Median)	p.value
Tumor size	pT1	1.375 ± 0.354 (1.275)	2.467 ± 1.207 (1.118)	0.681
	pT2	0.661 ± 0.221 (0.248)	2.029 ± 0.562 (1.458)	0.392
	pT3	0.537 ± 0.365 (0.229)	2.490 ± 1.346 (1.436)	0.043
Lymph nodes	N0	0.856 ± 0.236 (0.489)	1.932 ± 0.676 (0.491)	0.213
	N1	0.597 ± 0.332 (0.229)	1.437 ± 0.391 (1.452)	0.171
	N2	0.944 ± 0.440 (0.809)	4.626 ± 1.478 (4.753)	0.061
Tumor stage	I	1.015 ± 0.331 (1.148)	1.854 ± 0.922 (0.547)	0.491
	II	0.637 ± 0.250 (0.229)	1.688 ± .508 (0.997)	0.115
	III	0.814 ± 0.364 (0.298)	3.988 ± 0.508 (2.811)	0.042
Tumor grade	I	0.981 ± 0.427 (1.148)	3.176 ± 1.829 (2.811)	0.283
	II	0.856 ± 0.427 (1.148)	2.160 ± 0.562 (1.283)	0.051
	III	0.157 ± 0.085 (0.171)	1.590 ± 0.730 (1.436)	0.110

## Abbreviations

BC: Breast cancer
miR-26a: microRNA-26a-5p
HER-2: human epidermal growth factor receptor 2
EGFR: epidermal growth factor receptor
ADCC: antibody-dependent cell-mediated cytotoxicity
MTDH: metadherin
EZH2: enhancer of zeste homolog 2
MCL-1: myeloid cell leukemia 1
CCNE2: cyclin E2
FISH: fluorescence in situ hybridization
IHC: immunohistochemical
TNBC: triple-negative BC
PTEN: Prime time entertainment network
PDHX: Pyruvate Dehydrogenase Complex Component X
CHD1: Chromodomain-helicase-DNA-binding protein 1
GREB1: Growth regulation by estrogen in breast cancer 1
KPNA2: Karyopherin Alpha 2
LOH: loss of heterozygosity
EMT: epithelial–mesenchymal transition
